# What guidance exists to support patient partner compensation practices? A scoping review of available policies and guidelines

**DOI:** 10.1111/hex.13970

**Published:** 2024-01-18

**Authors:** Grace Fox, Dean A. Fergusson, Ahmed Sadeknury, Stuart G. Nicholls, Maureen Smith, Dawn Stacey, Manoj M. Lalu

**Affiliations:** ^1^ School of Epidemiology and Public Health University of Ottawa Ontario Ottawa Canada; ^2^ Clinical Epidemiology Program Ottawa Hospital Research Institute Ontario Ottawa Canada; ^3^ Department of Medicine University of Ottawa Ontario Ottawa Canada; ^4^ Office for Patient Engagement in Research Activities (OPERA) Ottawa Methods Centre, Ottawa Hospital Research Institute Ontario Ottawa Canada; ^5^ Patient Partner Ontario Ottawa Canada; ^6^ School of Nursing University of Ottawa Ontario Ottawa Canada; ^7^ Department of Cellular and Molecular Medicine University of Ottawa Ontario Ottawa Canada; ^8^ Department of Anesthesiology and Pain Medicine, The Ottawa Hospital University of Ottawa Ontario Ottawa Canada

**Keywords:** compensation, financial compensation, guidance, patient engagement, patient partner

## Abstract

**Background:**

An integral aspect of patient engagement in research, also known as patient and public involvement, is appropriately recognising patient partners for their contributions through compensation (e.g., coauthorship, honoraria). Despite known benefits to compensating patient partners, our previous work suggested compensation is rarely reported and researchers perceive a lack of guidance on this issue. To address this gap, we identified and summarised available guidance and policy documents for patient partner compensation.

**Methods:**

We conducted this scoping review in accordance with methods suggested by the JBI. We searched the grey literature (Google, Google Scholar) in March 2022 and Overton (an international database of policy documents) in April 2022. We included articles, guidance or policy documents regarding the compensation of patient partners for their research contributions. Two reviewers independently extracted and synthesised document characteristics and recommendations.

**Results:**

We identified 65 guidance or policy documents. Most documents were published in Canada (57%, *n* = 37) or the United Kingdom (26%, *n* = 17). The most common recommended methods of nonfinancial compensation were offering training opportunities to patient partners (40%, *n* = 26) and facilitating patient partner attendance at conferences (38%, *n* = 25). The majority of guidance documents (95%) suggested financially compensating (i.e., offering something of monetary value) patient partners for their research contributions. Across guidance documents, the recommended monetary value of financial compensation was relatively consistent and associated with the role played by patient partners and/or specific engagement activities. For instance, the median monetary value for obtaining patient partner feedback (i.e., consultation) was $19/h (USD) (range of $12–$50/h). We identified several documents that guide the compensation of specific populations, including youth and Indigenous peoples.

**Conclusion:**

Multiple publicly available resources exist to guide researchers, patient partners and institutions in developing tailored patient partner compensation strategies. Our findings challenge the perception that a lack of guidance hinders patient partner financial compensation. Future efforts should prioritise the effective implementation of these compensation strategies to ensure that patient partners are appropriately recognised.

**Patient or Public Contributions:**

The patient partner coauthor informed protocol development, identified data items, and interpreted findings.

## 1 INTRODUCTION

It is crucial to create a supportive and respectful environment for engagement in research. This helps ensure that all team members, including patient partners, can contribute fully to discussions and decisions.^1^ A specific approach to supporting patient partners is the provision of compensation (defined here as offering something of monetary value, goods or services in exchange for engagement; definitions can be found in Box 1).^8^ The compensation of patient partners for their contributions to health research is important for ethical and practical reasons.^2^, ^9^, ^10^ First, compensation demonstrates fairness. Researchers receive professional or academic recognition for their work, yet similar acknowledgements may not always be meaningful to patient partners. Compensation thus represents an opportunity to provide recognition appropriate to the patient context.^11^, ^12^, ^13^ Second, financial compensation can facilitate the participation of individuals who may not have the economic means to be engaged in research.^9^, ^12^ Third, compensation facilitates an inclusive environment that encourages patient partners to freely share their perspectives and maximises the impacts of their engagement.^10^

BOX 1. **Terminology used to describe patient engagement in research and methods of patient partner compensation.^2^, ^3^, ^4^, ^5^, ^6^, ^7^**

**Table:** 

Term	Definition
Reimbursement	Reimbursement of out‐of‐pocket expenses from engagement that are necessary to enable an individual to be engaged as a patient partner (travel, accommodations, parking, meals, child‐care support or personal health care devices such as supplemental oxygen for a plane trip).^2^
	Reimbursement is not a form of recognition/appreciation/compensation because patient partners should not pay out‐of‐pocket to be engaged in research.^2^
Patient partner	Individuals with lived experience of a health condition and informal caregivers, including family and friends.^5^
Patient engagement in research	The inclusion of patients as partners in the research process. Here, research is conducted ‘with’ patients, rather than ‘on’ patients. For example, patient partners can be actively engaged in governance, priority setting, developing the research questions and even performing certain parts of the research itself.^5^, ^6^
Compensation	Demonstrating appreciation of patient partner time, expertise and involvement in research as a partner. This includes offering something of monetary value, goods or services in exchange for engagement. Compensation can take on one of two forms: nonfinancial compensation and financial compensation.^3^, ^4^, ^7^
Nonfinancial compensation	Offering gifts, tokens of appreciation, opportunities or services in exchange for patient partnership on a research project. For example, this could be coauthorship on manuscripts or research material, facilitating patient partner attendance at a conference, education, or gifts (token of appreciation e.g., flowers, care package, gift card).^3^, ^4^, ^7^
Financial compensation	Financial compensation extends beyond the partner's reimbursement for out‐of‐pocket expenses and includes offering payment or something of monetary value in exchange for their engagement. For example, this could be honoraria, cash or salary (formal payroll).^3^, ^4^, ^7^
	Gifts or gift cards (for grocery stores, restaurants, retail stores, prepaid visa gift cards etc.) are considered financial compensation when the value is informed by a formal conversion (e.g., 2 h of work at 25$ per hour = $50 gift or gift card value) or patient partners decide that they want to receive payment in the form of gifts or gift cards.

Several organisations have now developed guidance documents to support the compensation (nonfinancial and financial) of patient partners in research. These include the National Institute for Health and Care Research in the United Kingdom, as well as the Strategy for Patient‐Oriented Research (SPOR) in Canada.^2^, ^14^, ^15^

Despite the availability of guidance, in a recent systematic review, we found that only a small fraction of studies reported offering financial compensation.^16^ In a follow‐up survey of researchers and their institutional representatives, participants reported a perceived lack of policy and guidance around compensation, both of which served as barriers to providing patient partner compensation. It thus appears that, despite known benefits to compensating patient partners, and the availability of guidance, awareness of guidance is limited. Alternatively, the available guidance may not be serving the needs of researchers to support the compensation of patient partners.

A synthesis of available guidance and policy documents would assist researchers in making informed decisions regarding the compensation of patient partners. It may also identify areas of inconsistency that could explain varied uptake and point to a need for further consolidated guidance. At present, no such synthesis of guidance exists.

To address this gap, we undertook a scoping review to identify and synthesise existing policies and guidance documents for patient partner compensation. Our overarching research question was, ‘What guidance or policies exist to inform patient partner compensation in research, and how do they compare?’

## 2 MATERIALS AND METHODS

We adhered to scoping review methods established by the JBI.^17^, ^18^ We also incorporated engagement with key groups, as described by Arksey and O'Malley,^19^ by collaborating with a patient partner and a multidisciplinary research team during the development, conduct and interpretation of the review and its findings. We registered the protocol on the Open Science Framework (https://osf.io/en8a4/) and published it as a part of a broader research programme.^16^ We have reported the review in accordance with the Preferred Reporting Items for Systematic Reviews and Meta‐Analyses extension for scoping reviews (PRISMA‐ScR).^20^ A complete PRISMA‐ScR checklist can be found in Supporting Information S1: Appendix 1.

### 2.1 Eligibility criteria

We used the *Types of Evidence Sources, Participants, Concept*, and *Context* framework to define our eligibility criteria.^18^ The *Types of Evidence Sources* included articles, documents or policies that provide information on processes or recommendations on patient partner compensation, but there were no restrictions on author type (e.g., organisations, research teams, patient partners). This represents an expanded approach from the published protocol,^7^ but was driven by a recognition that patient perspectives or experiences may not be reflected in official organisational or institutional documents. It also allowed us to cast a wide net and capture a range of perspectives on the topic of patient partner compensation. We excluded documents that solely reported activity or where the aim was not to provide guidance (e.g., meeting minutes, annual reports).

*Participants* were not applicable. However, the *Concept* was the compensation of patient partners in their role as research team members, consultants, or members of steering/advisory/grant review committees (i.e., patient partners). Patient partners were defined as individuals with lived experience of a health condition, including informal caregivers, members of the public, friends and family, who work with researchers to inform, develop or conduct research.^5^ We defined ‘compensation’ as offering goods or services, nonfinancial and financial, in exchange for engagement in research. We excluded documents that solely described compensation of patients for their role as research participants.

*Context* was patient engagement in health research, which refers to meaningful and active collaboration with patients in governance, priority setting, conduct or knowledge translation from a research activity.^1^ While we use the term patient engagement here, terms such as patient consultation or patient and public involvement may be used depending on jurisdiction.^21^, ^22^ There were no restrictions on the setting of patient engagement in research (e.g., clinical, health policymaking, preclinical) or stage of research (e.g., priority setting, study design, data collection, data analysis, dissemination).

### 2.2 Information sources and literature search

In line with expanding the scope of the review, we used additional searching strategies to our formal search of policy documents. Our previous systematic review identified 316 papers in which patient partner recognition was discussed; within this sample of papers 91% reported offering compensation to patient partners.^16^ From these 316 studies, we extracted any reported guidance or policy documents that were used to help authors develop a patient partner compensation strategy. These referenced guidance or policy documents were retrieved and reviewed against the present eligibility criteria for consideration of inclusion in the present scoping review. In addition, we included any institutional or policy guidance documents that were suggested as part of a survey of authors and institutions about compensation practices.^8^, ^16^ This initial corpus of documents was supplemented with a search of repositories for guidance and policy documents. We searched Overton.io, the largest international database of policy documents, on 5 April 2022.^23^ We piloted several search strategies with different variations of ‘patient engagement’ search terms before deciding on the term ‘patient partner’. Broader search terms (e.g., patient engagement, patient and public involvement) generated thousands of hits, the majority of which were not related to health research (i.e., health care decision‐making, informing system‐level processes). Additionally, we conducted searches of 17 international websites (Free, Subscription based, and Search Engines) listed in the Canadian Agency for Drugs and Technologies Grey Matters Tool^24^ (i.e., a tool used to guide searches of the grey literature). Finally, we searched Google and Google Scholar, collecting the first 50 hits for each of four text combinations (200 hits total for each search engine).^24^ All search strategies were developed in consultation with an information specialist (Lindsey Sikora, Head of Research Support [Health Sciences, Medicine, STEM], University of Ottawa), and are presented in Supporting Information S1: Appendix 2. We limited our searches to documents published after 2000 to maintain a contemporary focus. Searches were conducted on 29 March 2022. When we captured a source with vague information about patient partner compensation, an email was sent to the author asking for further information.

### 2.3 Selection of sources of evidence

All identified documents and hyperlinks were stored in a Microsoft Excel spreadsheet. Duplicates were removed and two independent reviewers (G. F. and A. S.) screened documents by full text. Reviewers participated in two piloting exercises screening 50 documents in total and resolving conflicts every 25 documents screened until an 80% agreement was achieved. All reasons for exclusion were recorded. Reviewers met to resolve conflicts and a third‐party reviewer (M. M. L. and D. A. F.) was consulted if the two reviewers could not reach consensus. When institutional policies or guidance documents were identified, we searched sources for the most up‐to‐date versions of documents before commencing data extraction.

### 2.4 Data charting

We uploaded the included documents to Distiller SR (Evidence Partners Incorporated), a cloud‐based software that supports reproducible work necessary for a scoping review. Two independent reviewers (G. F. and A. S.) extracted data using a standardised form with 59 data elements. Reviewers performed two pilot exercises on 10 documents until conflicts between reviewers were below five per document. Reviewers consulted a third party (M. M. L. and D. A. F.) if they could not reach a consensus.

### 2.5 Data items

We extracted document characteristics (e.g., source organisation, year of publication, country of origin, target audience), recommended methods of compensation (nonfinancial and financial methods), and financial compensation details (monetary amount, payment frequency). Gift cards were categorised as financial compensation when their value was explicitly tied to time provided and involved a formal calculation based on a pro‐rated amount (e.g., 2 h of work at $25/h = $50 gift or gift card value) or patient partners decide that they want to receive payment in the form of gift cards. In contrast, when gift cards were provided as a token of appreciation (i.e., no formal conversion took place) they were categorised as nonfinancial compensation. Additionally, we extracted (verbatim) items to consider when offering financial compensation as well as reported benefits, challenges, barriers, and enablers. All monetary amounts were converted to USD based on conversion rates on 5 September 2022. A complete list of data items can be found in Supporting Information S1: Appendix 3.

### 2.6 Synthesis of results

We presented document characteristics and recommendations (e.g., source document type, recommended methods of compensation, compensation details) descriptively. Two reviewers independently extracted verbatim statements of benefits, challenges, barriers and enablers to patient partner financial compensation. Following extraction, each reviewer independently read the extracted statements and inductively generated overarching themes (i.e., benefits, challenges, barriers, enablers and items to consider). All themes were tabulated and grouped through a process of data reduction. Any conflicts were resolved by reviewers. Overarching themes and frequency of reporting were presented to the entire team for feedback. We then narratively synthesized the overarching themes.

### 2.7 Patient engagement

A patient partner (M. S.) was engaged in this study and details are described following the Guidance for Reporting the Involvement of Patients and the Public short form (Supporting Information S1: Appendix 4). She informed protocol development and provided feedback on various aspects of the project, including data extraction and interpretation. We held regular monthly meetings to discuss the study progress and ensure that the patient's perspective was considered throughout.

We codeveloped a terms of reference a priori to document details of engagement (e.g., expectations, project goals). Our patient engagement plan was informed by INVOLVE's seven Core Principles of Engagement^25^ and the CIHR SPOR Patient Engagement framework.^1^ Recognition included coauthorship and financial compensation. Our financial compensation strategy was guided by the SPOR Evidence Alliance Patient Partner Appreciation Policy,^26^ which was discussed and approved by the patient partner. In addition to the patient partner, we also sought patient feedback by presenting to a hospital‐associated patient partner council.

## 3 RESULTS

### 3.1 Search results

**Figure 1:**
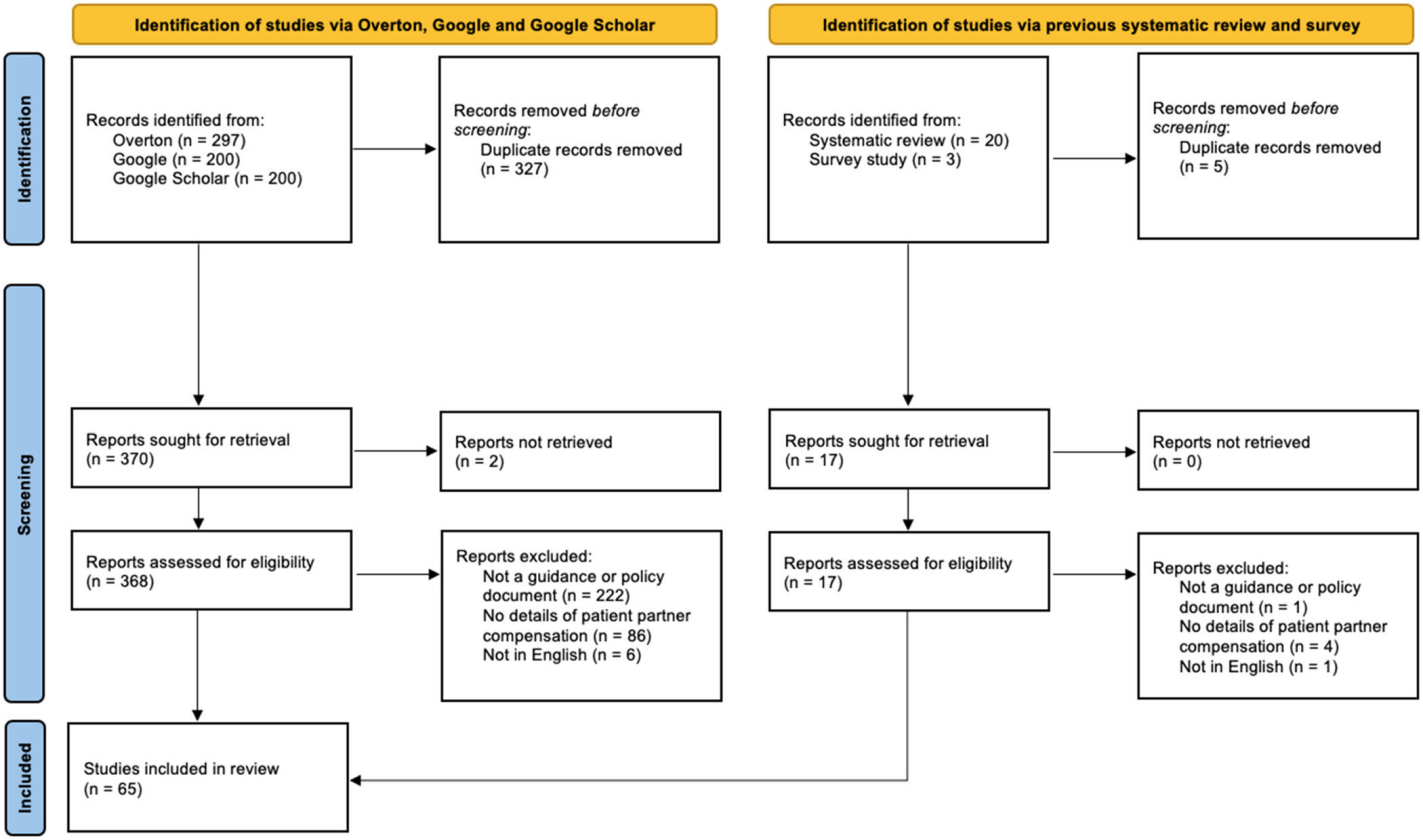
Preferred Reporting Items for Systematic Reviews and Meta‐Analyses flow diagram.

We screened 370 documents identified by the literature searches (Overton, Google, Google Scholar) and 17 documents identified by the previous systematic review and survey study. Sixty‐five documents met full eligibility criteria (54 from the literature search and 11 from previous studies) (Figure 1). A full list of identified documents can be found in Supporting Information S1: Appendix 5.

### 3.2 Document characteristics

Documents were published from 2012 to 2022, with the largest number published in 2021 (*n* = 16, 25%) (Table 1). The majority of documents were published in Canada (57%, *n* = 37) or the United Kingdom (26%, *n* = 17). The remaining documents originated from the United States (12%, *n* = 8), Switzerland (3%, *n* = 2) and Belgium (2%, *n* = 1). Most documents were developed by national organisations (51%, *n* = 33) and focused on providing guidance to researchers (54%, *n* = 35). A large proportion of documents were developed by research networks (45%, *n* = 29). All of the included documents were developed by public entities or nongovernmental organisations. Notably, 30 documents (46%) focused solely on patient partner compensation.

**Table 1:** Document characteristics (*n* = 65).

Document characteristic	*N*	%
Year of publication		
2012–2013	3	5%
2014–2015	1	2%
2016–2017	7	11%
2018–2019	8	12%
2020–2021	26	40%
2022	5	8%
Undated	15	23%
Country of origin		
Canada	37	57%
United Kingdom	17	26%
United States of America	8	12%
Switzerland	2	3%
Belgium	1	2%
Document source organisation (select all that apply)		
Research network	29	45%
Government organisation (i.e., agency established by national or provincial government)	15	23%
Health or academic institution	15	23%
Charity or foundation	6	9%
Nongovernmental organisations	4	6%
Government (e.g., Ministry of Health)	2	3%
Target audience (select all that apply)		
Researcher	35	54%
Patient partner	27	42%
Policy maker	9	14%
Researcher representative	5	8%
Industry member	3	5%
Unclear	15	23%
Level of policy‐making		
National	33	51%
Provincial or state‐specific	15	23%
Subprovincial/state	4	6%
International	3	5%
Unclear	10	15%
Document scope		
Focused on patient engagement with a section dedicated to compensation	30	46%
Focused on patient partner compensation	30	46%
General research guidance with a section on patient partner compensation	4	6%
Focused on compensation with a section dedicated to patient partners	1	2%

### 3.3 Recommended reimbursement practices and nonfinancial methods of compensation

The majority of documents recommend that patient partners be reimbursed for expenses associated with their engagement (89%, *n* = 58). Within these, key costs identified included: covering costs associated with conference attendance (43%, *n* = 25), babysitting/caregiver services (36%, *n* = 21) and accommodations (e.g., auditory or mobility impairments) (16%, *n* = 9). All documents recommended offering nonfinancial compensation to patient partners. Among all documents (*n* = 65) we identified 12 different suggested methods of nonfinancial compensation (Table 2). The two most common were offering training opportunities (40%, *n* = 26) and facilitating patient partner attendance at conferences (38%, *n* = 25).

**Table 2:** Nonfinancial and financial compensation details.

Document source and title	Nonfinancial compensation												Financial compensation				
	Services or training opportunities	Provide meals	Gift card^a^	Acknowledgement on research outputs	Verbal thanks	In‐kind donation	Gift	Conference presentation opportunity	Coauthorship	Invitation to a special event	Honorary appointment	Coinvestigator/coapplicant on grant	Honoraria	Gift card^b^	Salary	Stipend	Not specified^c^
South London and Maudsley NHS Foundation Trust (SLaM)—A guide to joining the Involvement Register																	x
Patient Voices Network, BC Patient Safety & Quality Council—A guide to patient engagement					x												
Patient Commando—A patient perspective on compensation	x						x						x				
NHS Birmingham Cross City Clinical Commissioning Group—Birmingham Cross City Clinical Commissioning Group Safeguarding Annual Report 2015/16																	
National Institute for Health Research, INVOLVE—Briefing notes for researchers: public involvement in NHS, public health and social care research				x				x									x
National Institute of Health Research, Mental Health Research Network and INVOLVE—Budgeting for involvement: Practical advice on budgeting for actively involving the public in research studies	x		x		x	x	x				x		x	x	x		
Canadian Venous Thromboembolism Research Network (CanVECTOR)—CanVECTOR Patient partners compensation policy													x				
The Canadian Donation and Transplantation Research Program (CDTRP)—CDTRP patient, family and donor partnership platform terms of reference													x				
National Institute for Health and Care Research—Centre for Engagement and Dissemination ‐ Recognition payments for public contributors	x	x				x							x				
Canadian Institutes of Health Research—Considerations when paying patient partners in research	x		x		x	x							x		x		
Canadian Institutes of Health Research (CIHR)—Ethics guidance for developing partnerships with patients and researchers		x					x	x									x
Clinical Trials Ontario—FAQ's						x							x				
Patient Advisors Network—FAQs about being involved as a patient partner		x				x							x	x	x		
IMAGINE—Financial compensation policy fro patient representatives																	x
NHS England—Framework for Patient and Public Participation in Public Health Commissioning	x			x	x												x
BC SUPPORT Unit—Fraser Centre Research Support Award in Patient‐Oriented Research												x					x
Richards—Guidance on authorship with and acknowledgement of patient partners in patient‐oriented research				x					x								
National Institute for Health Research NIHR Greater Manchester Patient Safety Translational Research Centre (Greater Manchester PSTRC)—Guidance on payments and expenses for non‐ Executive lay‐members of Executive Management Board		x											x				
CHILD‐BRIGHT—Guidelines for patient–partner compensation						x							x				
CanChild, Kids brain health network, McMaster University—Guidelines for researchers to complete research with family/patient partners including the SECRET to success	x												x	x			
ISPOR–ISPOR Code of Ethics 2017 (4th edition)																	x
BC Patient Safety and Quality Council–Mandate & terms of reference																	x
Aiyegbusi—Moving beyond project‐specific patient and public involvement in research	x				x								x				
Canadian Institute for Health Information—Participating in a CIHI Project Patient Toolkit													x			x	
CYSHCNet—Partnering with youth & families in research a standard of compensation for youth and family partners								x					x		x	x	
CYSHCNet Adult and Child Center for Outcomes Research and Delivery Science (ACCORDS)–Partnering with youth, families, & patients in research a standard of compensation for youth, family, and patient partners								x					x	x	x	x	
BC Mental Health and Substance Use Services (BCMHSUS)–Patient and family engagement framework																	x
BC Mental Health & Substance Use Services (BCMHSUS)—Patient and family partner handbook	x												x				
National Institute for Health Research Collaboration for Leadership in Applied Health Research and Care East of England–Patient and public involvement (PPI) in research handbook	x	x		x	x				x								x
Alberta SPOR SUPPORT Unit–Patient engagement in health research: a how‐to guide for patients													x				
Ontario SPOR SUPPORT Unit–Patient engagement in research: catalogue of organisations in Ontario													x				
Patvocates for Rising Tide Foundation–Patient involvement for applicants													x				
Caruncho—Patient‐oriented research in mental health: matching laboratory to life and beyond in Canada																	x
British Columbia Academic Health Science Network (BC AHSN)–Patient partner appreciation	x		x				x						x	x			
Newfoundland and Labrador's Support for People and Patient‐Oriented Research and Trials Unit–Patient partner appreciation–NL SUPPORT and quality of care NL guidelines	x			x	x		x						x		x		
Alberta Strategy for Patient‐Oriented Research (SPOR) SUPPORT Unit (AbSPORU) and the Patient Engagement Platform Compensation Working Group—Patient partner appreciation guidelines: compensation in research			x				x		x	x			x				
SPOR Evidence Alliance—Patient partner appreciation policy and protocol	x												x	x			
Maritime SPOR SUPPORT Unit (MSSU)—Patient partner compensation and reimbursement policy								x					x	x			
Richards—Patient partner compensation in research and health care: the patient perspective on why and how	x		x			x							x				
Saskatchewan Centre for Patient‐Oriented Research (SCPOR)—Patient partner honoraria													x				
Patient Voices Network, BC Patient Safety & Quality Council—Patient Partner, Lung Health Innovation Knowledge Translation Project																	x
Richards—Patients as partners in research: how to talk about compensation with patient partners	x		x	x					x				x				
Hoens—Patients as partners in research: there is plenty of help for researcher																	x
Smith—Patients as research partners; how to value their perceptions, contribution and labour?	x			x	x			x	x	x			x		x		
BC Mental Health & Substance Use Services (BCMHSUS)—Paying patient and family partners at BC mental health and substance use services a playbook on compensation	x												x	x	x		
National Institute for Health and Care Research—Payment guidance for researchers and professionals	x		x	x	x	x	x				x		x		x		
Centers for Medicare & Medicaid Services (CMS)—Person and Family Engagement Toolkit (PFE) a guide for measure developers													x				
National Institute for Health Research (NIHR) INVOLVE—Policy on payment of fees and expenses for members of the public actively involved with INVOLVE		x											x				
Belgian Health Care Knowledge Centre—Position of KCE on patient involvement in health care policy research	x	x					x						x	x			
The National Health Council—Principles for compensating patients for patient engagement activities	x																x
UCL Partners—Recognition payments for patients, carers and public contributors	x	x											x				
SPOR Networks in Chronic Diseases and the PICHI Network—Recommendations on patient engagement and compensation	x						x						x	x			
Rising Tide Foundation—Recommendations on patient involvement for funding institutions													x				
National Institute of Health Research INVOLVE—Reward and recognition for children and young people involved in research—things to consider	x			x				x		x				x			
National Institute for Health and Care Research—Reward and recognition for public contributors—a guide to the payment of fees and expenses	x	x				x							x				
Sepsis Canada—Sepsis Canada support request form (for members)																	x
The Change Foundation—Should money come into it? A tool for deciding whether to pay patient‐engagement participants													x				
US Department of Veteran Affairs—Strengthening Excellence in Research through Veteran Engagement (SERVE) Toolkit 2.0	x	x	x										x			x	
Wisconsin Department of Health Services—Team engagement for quality improvement welcome booklet		x	x														
Nuffield Department of Orthopaedics, Rheumatology and Musculoskeletal Sciences (NDORMS)—The role of the patient partner																	x
University of Calgary—Travel and expense reimbursement handbook											x						
Hamilton—Workbook to guide the development of a patient engagement in research (PEIR) plan	x			x			x	x	x				x				

^a^ Gift cards offered as a token of appreciation.

^b^ Gifts or gift cards (for grocery stores, restaurants, retail stores, prepaid visa gift cards etc.) are considered financial compensation when the value is informed by a formal conversion (i.e., 2 h of work at $25/h = $50 gift or gift card value) or patient partners decide that they want to receive payment in the form of gifts or gift cards.

^c^ Documents that recommend offering financial compensation, but do not recommend a specific method of offering financial compensation.

### 3.4 Recommended financial compensation methods

Sixty‐two documents (95%) recommended offering financial compensation to patient partners (Table 2). Suggested methods varied, with the most common being honoraria (69%, *n* = 43), gift cards (18%, *n* = 11), salary (15%, *n* = 9) or stipends (6%, *n* = 4). Nineteen documents (31%) did not suggest a specific method. Of note, no guidance documents advised against offering financial compensation to patient partners.

**Figure 2:**
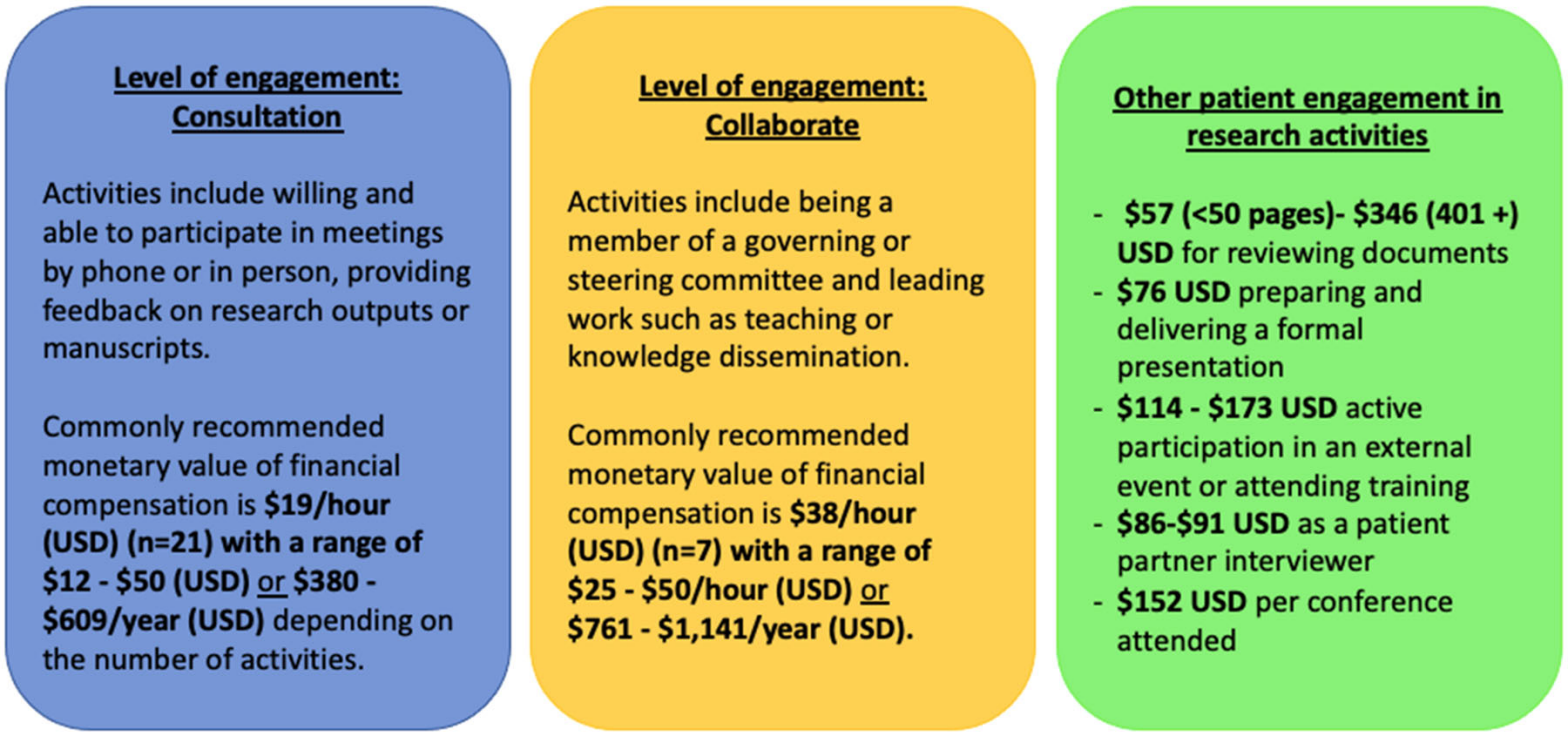
Comprehensive synthesis of financial compensation recommendations (i.e., monetary value) across guidance and policy documents. Further details can be found in Supporting Information S1: Appendix 5.

The recommended monetary value of financial compensation varied and was associated with the level of engagement and specific activities (Figure 2). Twenty‐one guidance documents suggested that patient partners should be offered a minimum of $19/h (USD) for ‘one‐time’ engagements or participating in consultation exercises, such as providing feedback on project proposals. In contrast, the median recommended monetary value for compensating patient partners holding positions on advisory committees was $38/h (USD). Additionally, one guidance document recommended using a Fair Market Value Calculator, which adjusts for patient partner expertise and experience, to determine the monetary value of financial compensation.^27^ Two organisations implemented caps on the annual income offered to patient partners ($228 and $1141 USD; Supporting Information S1: Appendix 6).^28^, ^29^

Seventeen documents (27%) provided guidance tailored to specific populations, including youth (*n* = 7), Indigenous peoples (*n* = 7), and individuals with disabilities (*n* = 3) (Supporting Information S1: Appendix 7). However, the majority of the included guidance focused on logistical aspects of financial compensation and the need to remain flexible to meet the needs of specific populations. For example, several documents highlighted the importance of offering cash to children or individuals affected by homelessness as they may not have bank accounts to deposit cheques.

### 3.5 Benefits, challenges, barriers and enablers to patient partner financial compensation

We identified reported benefits or challenges of financial compensation in 27 documents (Table 3). The two most commonly reported benefits were that financial compensation: (1) supports the inclusion of diverse perspectives by enabling individuals from different socioeconomic backgrounds to be engaged in research (*n* = 18); and (2) offers a tangible method to demonstrate patient partner appreciation (*n* = 8). Two key reported challenges to financial compensation were budgetary limitations of research projects (*n* = 5) and lack of institutional procedures (*n* = 5).

**Table 3:** Reported benefits, challenges, barriers and enablers of patient partner financial compensation.

Theme	Number of studies
Benefits (*n* = 23)	
Financial compensation supports the inclusion of diverse perspectives	18
Tangible method to demonstrate patient partner appreciation and supports a sense of equality among team members	8
Removes power imbalances among team members	4
Support patient partner commitment to the project and long‐term engagement	2
Benefits patient partners financially	3
Challenges (*n* = 5)	
Financial limitations and institutional procedures (e.g., patient engagement is not an eligible expense) can challenge ability to compensate patient partners	5
Financial payments can jeopardise disability or social security payments or impact income tax rates	3
Loss of autonomy associated with financial compensation	2

### 3.6 Items to consider when offering financial compensation to patient partners

We identified reported items to consider when offering or accepting financial compensation in 22 documents. The most commonly reported item was acknowledging that patient partners can refuse financial compensation or agree to accept less than what is offered (*n* = 19) (Supporting Information S1: Appendix 8). This highlights the importance of respecting individual preferences. Other commonly reported considerations included discussing compensation at the onset of the research project to foster transparent expectations of acknowledgement (*n* = 17). Fifteen documents highlighted that financial compensation for patient partners is classified as taxable income, which could not only impact income tax rates but also jeopardize patient partners' ability to collect other financial support (e.g., disability payments).

## 4 DISCUSSION

We identified and synthesised publicly accessible guidance documents on patient partner compensation, both nonfinancial and financial methods. All documents recommended offering nonfinancial compensation and we identified 12 unique methods including coauthorship, providing training opportunities and facilitating patient partner attendance at conferences. We also found consistent recommendations that patient partners need to be reimbursed for any expenses incurred from engagement, including travel or accommodations. The majority of guidance also suggested financially compensating patient partners for their contributions to research (e.g., through honoraria) and none advised against financial compensation. The recommended monetary value of compensation varied by organisation, with most recommending linking the amount to the specific role, level of engagement and time commitment of the patient partners. While conclusions from our previous systematic review^16^ and survey study^8^ highlighted lack of guidance as a key barrier to financial compensation, our scoping review findings counter this perceived notion by presenting several publicly available guidance documents. Importantly, none of the identified guidance or policy documents provided recommendations on how to implement guidance at an institutional level and rarely provided any details on how documents were developed.

Despite these areas of consensus, we observed discrepancies between documents that could present dilemmas for researchers seeking guidance. For example, the only guidance document that recommended using the Fair Market Value Calculator, which considers patient partner expertise and experience level to determine the monetary value of compensation, originated from the United States.^27^ However, six Canadian guidance documents recommended against such calculators and suggest that monetary value should reflect patient partner responsibility level and time commitment. These inconsistencies are also observed between guidance originating from the same country. For instance, we identified 17 Canadian guidance documents that provided specific recommendations for financial compensation. Twelve recommended offering patient partners $25 (CAD) per hour, two recommended offering patient partners $25–$50 (CAD) for participation in a half‐day meeting (up to 4 h of work), one recommended minimum wage ($15.50 [CAD]) per hour and the remaining two did not recommend specific monetary values. Similarly, Canadian guidance documents varied widely in recommendations for nonfinancial compensation. While it is reasonable that different research networks develop unique compensation guidance documents in their local context, there may be challenges associated with having various guidance documents. The negative impact of having too many choices has been supported by ‘the choice overload hypothesis’, which suggests that when individuals are presented with too many options, they may become overwhelmed and find it more difficult to make a decision.^30^

The scarcity of resources addressing compensation for patient partners from underrepresented groups could also contribute to the perception of a lack of guidance.^8^, ^16^ This is especially concerning as a key proposed benefit of financial compensation is the engagement of underrepresented populations. For example, in interviews with Indigenous patient partners and researchers regarding patient engagement in research, valuing patient partner contributions was identified as one of four key pillars to success.^31^ Specifically, compensation bolstered Indigenous patient partner confidence in being involved. However, the approach to offering financial compensation needs to be tailored to the patient partners engaged.^32^ In the case of Indigenous patient partners, one network of Canadian patient‐oriented organisations recommended to clarify that compensation is a gesture of appreciation rather than a transaction for their time.^4^ This is important as the latter could suggest the purchase of Indigenous knowledge, which cannot be commoditized by a person or institution.^4^ Despite these intricacies of partnering with specific populations, we identified a paucity of documents with guidance in this regard. At this time, we recommend that research teams carefully consider the distinct needs of diverse patient partner populations and codevelop compensation strategies that are attuned to their beliefs and requirements.

Notably, a few guidance documents discussed challenges to offering financial compensation to patient partners. One crucial item to consider (raised in only 15 documents originating from Canada and the United Kingdom) was the potential impact that financial compensation may have on existing income streams. Financial compensation, in the form of cheques or cash, is considered taxable income if the monetary amount exceeds a specific value. For instance, in Canada, compensation of $500 (CAD) or more per year is considered taxable income.^33^ Furthermore, if patient partners accept payment from engagement, it can interfere with eligibility for disability payments or sick leave. Additionally, receiving financial compensation may involve collection of personal information such as home address or social insurance number. Researchers must ensure that patient partners fully understand these implications of financial compensations to avoid exposing them to undue risk. Additional items to consider are outlined in Supporting Information S1: Appendix 8.

While our scoping review provides a comprehensive overview of available guidance around patient partner financial compensation, limitations must be noted. First, Overton is a relatively new database but, despite its novelty, evidence exists to support its validity and value in identifying relevant guidance documents.^34^ Additionally, we worked with an information specialist to verify our search strategies and supplemented the Overton search by searching the grey literature. Second, our search is limited to publicly available guidance and policy documents. Because of this, publicly inaccessible organisational or institutional policies are not accounted for. Lastly, literature searches were conducted several months ago and identified guidance may have been updated. The purpose of this review is to provide an overview of compensation recommendations, not to be used as a guidance document. Thus, we encourage researchers and institutions to identify the most recent versions of local guidance or policy when developing a compensation strategy.

## 5 CONCLUSION

We identified an abundance of publicly available documents to support the development of patient partner compensation strategies. This stands in contrast to our previous survey results that noted a perceived lack of guidance or policy to support patient partner compensation. This suggests that there may be a lack of awareness of existing guidance among researchers, or that existing guidance does not meet the needs of researchers. Future research to identify and address key barriers and challenges of patient partner compensation should be explored. All included documents recommended offering nonfinancial compensation to patient partners and the majority (95%) recommended offering financial compensation. Indeed, we did identify discrepancies and gaps that may contribute to a perceived lack of guidance on this issue. We suggest that our results underline a need to create consolidated guidance that identifies core items to consider in compensation strategies and should include consultation with patient partners to ensure that guidance responds to their needs and preferences. The identification of core items may help improve the implementation of patient partner compensation strategies across diverse research groups.

## AUTHOR CONTRIBUTIONS

**Grace Fox**: Conceptualisation; investigation; writing—original draft; writing—review and editing. **Dean A. Fergusson**: Conceptualisation; writing—review and editing; resources; supervision. **Ahmed Sadeknury**: Writing—review and editing; investigation. **Stuart G. Nicholls**: Writing—review and editing. **Maureen Smith**: Writing—review and editing. **Dawn Stacey**: Writing—review and editing. **Manoj M. Lalu**: Conceptualisation; writing—original draft; writing—review and editing; supervision; resources.

## CONFLICT OF INTEREST STATEMENT

The authors declare no conflicts of interest.

## Supporting information

Supporting information.

## Data Availability

Data sharing is not applicable to this article as no new data were created or analysed in this study. All data generated or analysed during this study are included in this published article and its Supporting Information files.
