# Supplementary material for: What guidance exists to support patient partner compensation practices? A scoping review of available policies and guidelines
Source: Health Expect. 2024 Jan 18;27(1):e13970. doi: 10.1111/hex.13970 (PMC10795087; doi:10.1111/hex.13970)
Supplement: Supplementary file 1 — Supporting information. [file HEX-27-e13970-s001.docx]

**APPENDIX**

**Appendix 1. Complete PRISMA-ScR checklist**(36)

| **SECTION** | **ITEM** | **PRISMA-ScR CHECKLIST ITEM** | **REPORTED ON PAGE #** |
| --- | --- | --- | --- |
| **TITLE** | | | |
| Title | 1 | Identify the report as a scoping review. | 1 |
| **ABSTRACT** | | | |
| Structured summary | 2 | Provide a structured summary that includes (as applicable): background, objectives, eligibility criteria, sources of evidence, charting methods, results, and conclusions that relate to the review questions and objectives. | 3 |
| **INTRODUCTION** | | | |
| Rationale | 3 | Describe the rationale for the review in the context of what is already known. Explain why the review questions/objectives lend themselves to a scoping review approach. | 4 |
| Objectives | 4 | Provide an explicit statement of the questions and objectives being addressed with reference to their key elements (e.g., population or participants, concepts, and context) or other relevant key elements used to conceptualize the review questions and/or objectives. | 5 |
| **METHODS** | | | |
| Protocol and registration | 5 | Indicate whether a review protocol exists; state if and where it can be accessed (e.g., a Web address); and if available, provide registration information, including the registration number. | 6 |
| Eligibility criteria | 6 | Specify characteristics of the sources of evidence used as eligibility criteria (e.g., years considered, language, and publication status), and provide a rationale. | 6-7 |
| Information sources* | 7 | Describe all information sources in the search (e.g., databases with dates of coverage and contact with authors to identify additional sources), as well as the date the most recent search was executed. | 7 |
| Search | 8 | Present the full electronic search strategy for at least 1 database, including any limits used, such that it could be repeated. | 7 |
| Selection of sources of evidence† | 9 | State the process for selecting sources of evidence (i.e., screening and eligibility) included in the scoping review. | 7-8 |
| Data charting process‡ | 10 | Describe the methods of charting data from the included sources of evidence (e.g., calibrated forms or forms that have been tested by the team before their use, and whether data charting was done independently or in duplicate) and any processes for obtaining and confirming data from investigators. | 8 |
| Data items | 11 | List and define all variables for which data were sought and any assumptions and simplifications made. | 8, 30 |
| Critical appraisal of individual sources of evidence§ | 12 | If done, provide a rationale for conducting a critical appraisal of included sources of evidence; describe the methods used and how this information was used in any data synthesis (if appropriate). | N/A |
| Synthesis of results | 13 | Describe the methods of handling and summarizing the data that were charted. | 8 |
| **RESULTS** | | | |
| Selection of sources of evidence | 14 | Give numbers of sources of evidence screened, assessed for eligibility, and included in the review, with reasons for exclusions at each stage, ideally using a flow diagram. | 15 |
| Characteristics of sources of evidence | 15 | For each source of evidence, present characteristics for which data were charted and provide the citations. | 9-11, 16-21 |
| Critical appraisal within sources of evidence | 16 | If done, present data on critical appraisal of included sources of evidence (see item 12). | N/A |
| Results of individual sources of evidence | 17 | For each included source of evidence, present the relevant data that were charted that relate to the review questions and objectives. | 9-11, 16-21 |
| Synthesis of results | 18 | Summarize and/or present the charting results as they relate to the review questions and objectives. | 9-11 |
| **DISCUSSION** | | | |
| Summary of evidence | 19 | Summarize the main results (including an overview of concepts, themes, and types of evidence available), link to the review questions and objectives, and consider the relevance to key groups. | 11-13 |
| Limitations | 20 | Discuss the limitations of the scoping review process. | 14 |
| Conclusions | 21 | Provide a general interpretation of the results with respect to the review questions and objectives, as well as potential implications and/or next steps. | 1 |
| **FUNDING** | | | |
| Funding | 22 | Describe sources of funding for the included sources of evidence, as well as sources of funding for the scoping review. Describe the role of the funders of the scoping review. | 2 |

**Appendix 2. Search strategies**

| **Search source** | **Search strategies** |
| --- | --- |
| Overton.io | “patient partner” AND “research” AND “compensate*” |
|  | “patient partner” AND “research” AND “remunerat*” |
|  | “patient partner” AND “research” AND “pay*” |
|  | “patient partner” AND “research” AND “reimburse*” |
| Google | “patient partner” AND “research” AND “compensate*” |
|  | “patient partner” AND “research” AND “remunerat*” |
|  | “patient partner” AND “research” AND “pay*” |
|  | “patient partner” AND “research” AND “reimburse*” |
| Google Scholar | “patient partner” AND “research” AND “compensate*” |
|  | “patient partner” AND “research” AND “remunerat*” |
|  | “patient partner” AND “research” AND “pay*” |
|  | “patient partner” AND “research” AND “reimburse*” |

**Appendix 3. Data items**

| Data item |
| --- |
| 1. Organization/author name and contact information 2. Type of organization (Select all that apply: Government, Government organization, Research Network, Health or Academic institution, Charity or Foundation, Industry, Non-governmental organization) 3. Year of publication 4. Country of origin 5. Publication title 6. Target audience (Select all that apply: researchers, patient partners, researcher representative, industry member, policy maker) 7. Population specifics (Select all that apply: individuals with chronic conditions, youth and children, individuals with disabilities, seldom heard populations, Indigenous peoples) 8. What level of policymaking is the document supporting? (Select one: sub provincial/state, provincial/state, national supra-national) 9. Scope of the document (Select one: focused on patient partner compensation, Focused on patient engagement with a section dedicated to compensation, Focused on compensation with a section dedicated to patient partners, Focused on research guidance with a section on patient partner compensation) 10. Recommended methods of compensation (non-financial and financial) (Select all that apply) 11. Details of financial compensation (e.g., type of payment, frequency, amount etc.) 12. Reported benefits or challenges of patient partner compensation (Open text) 13. Reported barriers or enablers to guidance document implementation (Open text) 14. Special considerations when compensating patient partners (Open text) |

**Appendix 4. GRIPP II short-form**

| **Section and topic** | **Item** |
| --- | --- |
| **1: Aim** | Conduct a scoping review to identify guidance and policy documents aiming to guide patient partner compensation (non-financial and financial methods). To partner with a patient partner throughout the development and conduct of the scoping review. |
| **2: Methods** | One patient partner (MS) was recruited to join the research team through personal referral. MS was involved in developing the protocol, defining compensation terms, analyzing scoping review results and contributed to edits of this paper. MS attended virtual team meetings and continued to meet with GF monthly. MS was offered financial compensation and co-authorship in recognition of her contributions to the research project. |
| **3: Results** | Patient engagement contributed to the study in several ways including:   - Informing the project proposal with the patient partner experience: MS is well integrated in the patient engagement field and has a wealth of experience being a patient partner for several organizations. MS has experience with various methods of compensation, barriers to financial compensation and the different perspectives that patient partners have on financial compensation. - Provided insight into experiences working with international institutional institutes and their compensation practices. - Analyzing themes from the thematic analysis - Reviewed and edited this manuscript |
| **4: Discussion** | Overall, patient engagement was successful in informing scoping review development and conduct. Additionally, the research team learned a lot about the patient partner experience with financial compensation and how institutions are recognizing patient partners for their expertise through discussions with MS about her unique experiences. It was helpful that MS was familiar with most team members before joining the research team and that members of the team had experience with patient engagement.  The scoping review was conducted within a year and alongside two additional research projects. At the beginning of the project, we co-developed a timeline and budget to reflect the number of hours that MS devoted to the project. In the future, we will refer back to this timeline at the mid-term mark to ensure that the number of hours budgeted for were accurate. |
| **5: Reflections** | Engagement was embedded within the research project and MS was a member of the research team. Not only was engagement successful in shaping the scoping review, but it was also personally rewarding to have the opportunity to work closely with a person with lived experience. |

**Appendix 5. Full list of included documents**

1. Center for Patient Partnerships, Wisconsin CYSHCN Program and Partners. Team Engagement for Quality Improvement Welcome Booklet [Internet]. 2018. Available from: https://www.dhs.wisconsin.gov/publications/p02349.pdf

2. US Department of Veteran Affairs. SERVE Toolkit for Veteran Engagement: Planning [Internet]. Available from: https://www.hsrd.research.va.gov/for_researchers/serve/Section1-Planning.pdf

3. University of Calgary. Travel and Expense Reimbursement Handbook [Internet]. Available from: https://www.ucalgary.ca/finance/sites/default/files/teams/5/ap-travel-and-expense-reimbursement-handbook.pdf

4. UCL Partners. Recognition payments for patients, carers and public contributors. 2022.

5. The National Health Council. Principles for Compensating Patients for Patient Engagement Activities [Internet]. 2021. Available from: https://nationalhealthcouncil.org/wp-content/uploads/2021/06/NHC-_FMV_Patient-Engagement-Compensation_Prinicples-final_.pdf

6. Strategy for Patient-Oriented Research (SPOR). Compensation Guidelines for Engaging Patients as Partners in Research [Internet]. 2017. Available from: https://diabetesaction.ca/wp-content/uploads/2018/02/CIHR-DRAFT-SPOR-Compensation-Guidelines-for-Patient-Partners-in-Research-draft_CLEAN.pdf

7. SPOR Evidence Alliance (2019). Patient Partner Appreciation Policy and Protocol. Toronto, ON: SPOR Evidence Alliance.

8. South London and Maudsley NHS Foundation Trust (SLaM ). A Guide to joining the Involvement Register [Internet]. Available from: https://slamtwigops.files.wordpress.com/2011/10/involvement-register-leaflet-all-you-need-to-know.pdf

9. Request for Support [Internet]. Sepsis Canada. [cited 2022 Sep 20]. Available from: https://www.sepsiscanada.ca/resources/request-for-support?resourceID=491

10. Saskatchewan Centre for Patient-Oriented Research (SCPOR). Patient Partner Honoraria [Internet]. 2021. Available from: https://static1.squarespace.com/static/5c869fd0e666695abe893b3b/t/60d1f784bb7dc2611e55d724/1624373124153/patient+partner+honoraria_June+15.pdf

11. Patvocates for Rising Tide Foundation. Recommendations on Patient Involvement for Funding Institutions [Internet]. 2021. Available from: https://www.risingtide-foundation.org/fileadmin/CCR/Program/2021_06_22_Recommendations_on_Patient_Involvement_for_Funding_Institutions_v1.5.pdf

12. Patvocates for Rising Tide Foundation. Patient Involvement for Applicants [Internet]. 2021. Available from: https://www.risingtide-foundation.org/fileadmin/CCR/Program/2021_06_22_Patient_Involvement_for_Applicants_v1.5.pdf

13. Patient Voices Network, BC Patient Safety & Quality Council. A Guide to Patient Engagement [Internet]. Available from: https://patientvoicesbc.ca/wp-content/uploads/2019/07/PVN_Getting-Started-with-Patient-Engagement_WEB.pdf

14. Press Z. A Patient Perspective on Compensation.

15. Members | Patient Advisors Network [Internet]. 2021 [cited 2022 Sep 20]. Available from: https://www.patientadvisors.ca/members/

16. Patient Engagement in Research: Catalogue of Organizations in Ontario [Internet]. Clinical Trials Ontario. [cited 2022 Sep 20]. Available from: https://www.ctontario.ca/patients-public/resources-for-engaging-patients/patient-engagement-in-research-catalogue-of-organizations-in-ontario/

17. The Role of the Patient Partner — Nuffield Department of Orthopaedics, Rheumatology and Musculoskeletal Sciences [Internet]. [cited 2022 Sep 20]. Available from: https://www.ndorms.ox.ac.uk/get-involved/ndorms-patient-public-involvement-group-1/the-role-of-the-patient-partner

18. NHS England. NHS England Patient and Public Voice Partners Policy [Internet]. 2017. Available from: https://www.england.nhs.uk/wp-content/uploads/2017/08/patient-and-public-voice-partners-policy-july-2017.pdf

19. NHS. Working with our patient and public voices (PPV) partners Reimbursing expenses and paying involvement payments [Internet]. 2021. Available from: https://www.england.nhs.uk/wp-content/uploads/2017/08/B0869_Working-with-patient-and-public-voice-partners-reimbursing-expenses-and-paying-involvement-payments.pdf

20. NHS England. Framework for patient and public participation in public health commissioning [Internet]. Available from: https://www.england.nhs.uk/wp-content/uploads/2017/01/ph-participation-frmwrk.pdf

21. NHS Birmingham Cross City Clinical Commissioning Group. Strategic Patient Partner – proposed role profile. 2016.

22. NHS Barnsley Clinical, Commissioning Group. Patient and Public Involvement Strategy 2019 – 2021.

23. Newfoundland and Labrador’s Support for People and Patient-Oriented Research and Trials Unit. Patient Partner Appreciation – NL SUPPORT and Quality of Care NL Guidelines [Internet]. 2021. Available from: https://nlsupport.ca/wp-content/uploads/2022/07/NL_SUPPORT_Patient_Partner_Appreciation_2022.pdf

24. Mental Health Research Network and INVOLVE (2013) Budgeting for involvement: Practical advice on budgeting for actively involving the public in research studies, Mental Health Research Network, London and INVOLVE, Eastleigh.

25. INVOLVE. Reward and recognition for children and young people involved in research – things to consider [Internet]. 2016. Available from: https://www.invo.org.uk/wp-content/uploads/2016/04/INVOLVECYPrewardandrecognitionFinalApril2016.pdf

26. INVOLVE. Briefing notes for researchers: involving the public in NHS, public health and social care research [Internet]. 2012. Available from: https://www.invo.org.uk/wp-content/uploads/2014/11/9938_INVOLVE_Briefing_Notes_WEB.pdf

27. NIHR Greater Manchester Patient Safety Translational Research Centre (Greater Manchester PSTRC). Guidance on payments and expenses for nonExecutive lay-members of Executive Management Board [Internet]. Available from: http://www.patientsafety.manchester.ac.uk/media/mhs/bmh-faculty/gmpstrc/PSTRC-Payments-Policy-October-2017.pdf

28. National Institute for Health Research. Patient and Public Involvement (PPI) in research handbook. [Internet]. 2017. Available from: https://www.clahrc-eoe.nihr.ac.uk/wp-content/uploads/2018/01/CLAHRC-EoE-PPI-IN-Research-Handbook_December-2017.pdf

29. INVOLVE. Policy on payment of fees and expenses for members of the public actively involved with INVOLVE [Internet]. 2016. Available from: https://www.invo.org.uk/wp-content/uploads/2016/05/INVOLVE-internal-payment-policy-2016-final-1.pdf

30. INVOLVE. Developing training and support for public involvement in research [Internet]. 2012. Available from: https://www.invo.org.uk/wp-content/uploads/2012/11/INVOLVETrainingSupport2012.pdf

31. Reward and recognition for public contributors - a guide to the payment of fees and expenses [Internet]. [cited 2022 Sep 20]. Available from: https://www.nihr.ac.uk/documents/reward-and-recognition-for-public-contributors-a-guide-to-the-payment-of-fees-and-expenses/12248

32. Centre for Engagement and Dissemination - Recognition payments for public contributors [Internet]. [cited 2022 Sep 20]. Available from: https://www.nihr.ac.uk/documents/centre-for-engagement-and-dissemination-recognition-payments-for-public-contributors/24979

33. Payment guidance for researchers and professionals [Internet]. [cited 2022 Sep 20]. Available from: https://www.nihr.ac.uk/documents/payment-guidance-for-researchers-and-professionals/27392

34. Johnston JN, Ridgway L, Cary-Barnard S, Allen J, Sanchez-Lafuente CL, Reive B, et al. Patient oriented research in mental health: matching laboratory to life and beyond in Canada. Research Involvement and Engagement. 2021 Apr 26;7(1):21.

35. Turner G, Aiyegbusi OL, Price G, Skrybant M, Calvert M. Moving beyond project-specific patient and public involvement in research. J R Soc Med. 2020 Jan;113(1):16–23.

36. Hoens AM, Belton J, Scott A, Ardern CL. Patients as Partners in Research: There Is Plenty of Help for Researchers. Journal of Orthopaedic & Sports Physical Therapy. 2020 May;50(5):219–21.

37. Hamilton CB, Hoens AM, Backman CL, English K, McKinnon AM, McQuitty S, et al. Workbook to guide the development of a Patient Engagement In Research (PEIR) Plan [Internet]. 2018. Available from: https://www.arthritisresearch.ca/wp-content/uploads/2018/06/PEIR-Plan-Guide.pdf

38. 2018. Available from: https://www.arthritisresearch.ca/wp-content/uploads/2018/06/PEIR-Plan-Guide.pdf

39. Richards DP, Jordan I, Strain K, Press Z. Patients as Partners in Research: How to Talk About Compensation With Patient Partners. J Orthop Sports Phys Ther. 2020 Aug;50(8):413–4.

40. Maritime SPOR SUPPORT Unit (MSSU). Patient Partner Compensation and Reimbursement Policy [Internet]. 2020. Available from: https://mssu.ca/wp-content/uploads/2021/10/Eng-Patient-Partner-Compensation-Policy-May-15-2020-final.pdf

41. Santos J, Palumbo F, Molsen-David E, Willke RJ, Binder L, Drummond M, et al. ISPOR Code of Ethics 2017 (4th Edition). Value in Health. 2017 Dec;20(10):1227–42.

42. CYSHCNet Adult and Child Center for Outcomes Research and Delivery Science (ACCORDS). Partnering with Youth, Families, & Patients in Research A Standard of Compensation for Youth, Family, and Patient Partners [Internet]. 2021. Available from: https://cyshcnet.org/wp-content/uploads/2021/09/Standard-of-Compensation-2021-Partners.pdf

43. Introduction [Internet]. Clinical Trials Ontario. [cited 2022 Sep 20]. Available from: https://www.ctontario.ca/patients-public/resources-for-engaging-patients/patient-decision-aid/introduction/

44. Centers for Medicare & Medicaid Services (CMS). Person and Family Engagement Toolkit (PFE) A Guide for Measure Developers. 2021.

45. Boyer L, Libralesso P, Pot S. Guidelines for researchers to complete research with family/patient partners including the SECRET to success [Internet]. Available from: https://canchild.ca/system/tenon/assets/attachments/000/003/538/original/Guidelines_for_researchers.pdf

46. Participating in a CIHI Project — Patient Toolkit. :21.

47. British Columbia Academic Health Science Network (BC AHSN). Patient Partner Appreciation. 2021.

48. Cleemput Irina, Dauvrin Marie, Kohn Laurence, Mistiaen Patriek, Christiaens Wendy, Léonard Christian. Position of KCE on patient involvement in health care policy research . Method. Brussels. Belgian Health Care Knowledge Centre (KCE). 2019. KCE Reports 320. .

49. BC SUPPORT Unit. Fraser Centre Research Support Award in Patient-Oriented Research [Internet]. Available from: https://www.fraserhealth.ca/-/media/Project/FraserHealth/FraserHealth/Health-Professionals/Research-and-Evaluation-Services/Research-Support-Award-Guidelines_final.pdf

50. Mandate & Terms of Reference / BC Patient Safety & Quality Council [Internet]. BC Patient Safety & Quality Council. [cited 2022 Sep 20]. Available from: https://bcpsqc.ca/about-the-council/terms-of-reference/

51. BC Mental Health and Substance Use Services (BCMHSUS). Patient and Family Engagement Framework [Internet]. Available from: http://www.bcmhsus.ca/allpageholding/Documents/BCMHSUS%20Patient%20and%20Family%20Engagement%20Framework.pdf

52. BC Mental Health & Substance Use Services (BCMHSUS). Patient and Family Partner Handbook [Internet]. Available from: http://www.bcmhsus.ca/allpageholding/Documents/Patient%20and%20Family%20Partner%20Handbook%20FINAL.PDF

53. BC Mental Health & Substance Use Services (BCMHSUS). Paying Patient and Family Partners at BC Mental Health and Substance Use Services A Playbook on Compensation [Internet]. 2021. Available from: http://www.bcmhsus.ca/allpageholding/Documents/Compensation%20Guidelines_Ver%203.pdf

54. Alberta SPOR SUPPORT Unit Patient, Engagement Platform Compensation Working Group, Alberta SPOR SUPPORT Unit. Patient Partner Appreciation Guidelines: Compensation in Research [Internet]. Available from: https://absporu.ca/wp-content/uploads/2020/11/Albertans4HealthResearch_Appreciation-Guidelines_Oct-2019_V6.0.pdf

55. Richards DP, Birnie KA, Eubanks K, Lane T, Linkiewich D, Singer L, et al. Guidance on authorship with and acknowledgement of patient partners in patient-oriented research. Research Involvement and Engagement [Internet]. 2020;6(1). Available from: https://www.scopus.com/inward/record.uri?eid=2-s2.0-85089605386&doi=10.1186%2fs40900-020-00213-6&partnerID=40&md5=5e99d1de8a6ea71ec302950b83195605 https://researchinvolvement.biomedcentral.com/track/pdf/10.1186/s40900-020-00213-6.pdf

56. The Canadian Donation and Transplantation Research Program (CDTRP). (2021) CDTRP Patient, Family and Donor Partnership Platform Terms of Reference. https://cdtrp.ca/wp-content/uploads/2021/02/PFD-Terms-of-Reference-2Feb2021.pdf.

57. Smith E, Bélisle-Pipon JC, Resnik D. Patients as Research Partners; How to Value their Perceptions, Contribution and Labor? Citizen Science: Theory and Practice. 2019 Mar 8;4(1):15.

58. CHILD-BRIGHT. Guidelines for Patient-Partner Compensation. 2020.

59. Canadian Venous Thromboembolism Research Network (CanVECTOR). CanVECTOR Patient Partners Compensation Policy [Internet]. [cited 2022 Jul 19]. Available from: https://www.canvector.ca/platforms/patient-partners/canvector-pp-compensation-policy_v3-approved-march-2021.pdf

60. The Change Foundation. Should money come into it? A tool for deciding whether to pay patient-engagement participants [Internet]. 2015 [cited 2022 Jul 19]. Available from: https://ontariocaregiver.ca/wp-content/uploads/2021/06/Should-money-come-into-it.pdf

61. Alberta SPOR SUPPORT Unit. Patient Engagement in Health Research: A How-to Guide for Researchers [Internet]. 2018 [cited 2022 Jul 18]. Available from: https://albertainnovates.ca/app/uploads/2018/06/How-To-Guide-Researcher-Version-8.0-May-2018.pdf

62. Government of Canada CI of HR. Draft Ethics Guidance for Developing Research Partnerships with Patients - For public consultation - CIHR [Internet]. 2018 [cited 2022 Jul 14]. Available from: https://cihr-irsc.gc.ca/e/51226.html

63. Government of Canada CI of HR. Considerations when paying patient partners in research - CIHR [Internet]. 2019 [cited 2022 Jun 15]. Available from: https://cihr-irsc.gc.ca/e/51466.html

64. SPOR Networks in Chronic Diseases and the PICHI Network. Recommendations on Patient Engagement Compensation [Internet]. 2018. Available from: https://diabetesaction.ca/wp-content/uploads/2018/07/TASK-FORCE-IN-PATIENT-ENGAGEMENT-COMPENSATION-REPORT_FINAL-1.pdf

65. Richards DP, Jordan I, Strain K, Press Z. Patient partner compensation in research and health care: the patient perspective on why and how. Patient Experience Journal. 2018;5(3).

**Appendix 6. Recommended monetary values and frequency of financial compensation payments**

| Organization name | Monetary amount | Payment frequency |
| --- | --- | --- |
| Alberta Strategy for Patient-Oriented Research (SPOR) SUPPORT Unit (AbSPORU) and the Patient Engagement Platform Compensation Working Group. | $25/hour (CAD) ($19/hour (USD)) (Consult-Level)]  < 4 meetings/yr between $100-$200 (CAD) ($76 - $152 (USD)) [Committee members on a standing working group (Involve-Level)]  > 4 meetings/yr between $200-$400 (CAD) ($152 – $305 (USD)) [Committee members on a standing working group (Involve-Level)] | Pay per activity completed or meeting attended  One payment per year |
| BC Mental Health & Substance Use Services (BCMHSUS) | $25.00/hour (CAD) ($19/hour (USD)) [serving in an advisory and consultative role.]  $30.00/hour (CAD) ($23/hour (USD)) [developing literature based on their lived experience.]  $50.00/hour(CAD) ($38/hour (USD)) [leading work, such as teaching and knowledge dissemination.] | N/R |
| BC SUPPORT Unit | Studies awarded funding can allocate $500 (CAD) ($380 (USD)) to cover patient engagement expenses. | N/R |
| British Columbia Academic Health Science Network (BC AHSN) | $25-75 (CAD) ($19 - $57 (USD)) [One-time engagement of less than half-day]  $50-75 (CAD) ($38 - $57 (USD)) [One-time engagement of half day (+/- 4 hours)]  $75-150 (CAD) ($57 - $114 (USD)) [One-time engagement of full day (+/- 8 hours)]  $150-300 (CAD) ($114 - $228 (USD)) [Committee or working group that meets <= 6 times per year]  $ 300-500 (CAD) ($228 - $380 (USD)) [Committee or working group that meets between 7 and 12 times per year]  $500-1000 ($380 - $761 (USD)) [Committee or working group that meets > 12 times per year] | Pay per activity completed or meeting attended  One payment per year |
| Canadian Venous Thromboembolism Research Network (CanVECTOR) | $50/hour (CAD) ($38/hour (USD) [1 to 4 total hour commitments]  $200 per conference attended (CAD) ($152 (USD)) | Quarterly |
| CHILD-BRIGHT | $500.00 per year (CAD) ($380 per year (USD)) [Availability by email; willing and able to participate in a few meetings by phone or in person]  $1,000.00 per year (CAD) ($761 per year (USD)) [Commitment to a committee (includes meetings, follow-up actions, etc.)]  $1,000.00 - $1,500.00 per year (CAD) ($761 - $1,141 (USD)) [Contributing member in a governing committee (includes meetings, follow-up actions, etc.)]  $100.00 per event (CAD) ($76 per event (USD)) [Preparation and delivery of formal presentation, either in person or via webinar after working with network organizers to ensure alignment with meeting’s or webinar’s objectives]  $50.00 per event (CAD) ($38 per event (USD)) [Participation in informal panel or facilitation of small group]  $75.00 per ½ day, $150.00 per day (CAD) ($57 per ½ day, $114 per day (USD)) [Active participation at external event as a patient- partner]  $75.00 per ½ day, $150.00 per day (CAD) ($57 per ½ day, $114 per day (USD)) [Attendance or Attendance and Completion of training]  Patient partners will receive compensation of $100 per year (CAD) ($76 per year (USD)) in addition to the compensation they receive for their contributions as members of research teams or network committees. | Semi-annual payments |
| Clinical Trials Ontario | $25/hour (CAD) ($19/hour (USD))  $500 to $800 per year (CAD) ($380 to $609 per year (USD)), depending on number of meetings and other requirements | N/R |
| Children and Youth with Special Health Care Needs National Research Network (CYSHCNet) | Payments begin at a rate of $25 per hour (CAD) ($19/hour (USD)) with a $100 (CAD) minimum payment. ($76 (USD)) | N/R |
| IMAGINE | $25/hr (CAD) ($19/hour (USD)) | N/R |
| Maritime SPOR SUPPORT Unit (MSSU) | $25 - $50 (CAD) ($19 - $57 (USD)) [One time engagement of less than one half- day (less than 4 hours)  $50 - $75 (CAD) ($38 - $57 (USD)) [One time engagement of a half day (4 - 5 hours)]  $75 - $100 (CAD) ($57 - $76 (USD)) [Full day engagement (approximately 8 hours)]  $300 - $400 (CAD) ($228 - $304 (USD)) [Standing committee or working group that meets 8-12 times per year]  $100 - $300 (CAD) ($57 - $228 (USD)) [Standing committee or working group that meets less than 8 times per year.] | Pay per activity completed or meeting attended  One payment per year  Semi-annual payments |
| Richards, D | $25/hour (CAD) ($19/hour (USD))  $50/meeting per month (CAD) ($38/meeting per month (USD)) [personal experience]  $250 (CAD) ($190 (USD)) [a daily committee fee or daily research work fee]  $500-800/year (CAD) ($380 – $609/year (USD)) [partner in a specific research project]  $1,000-1,200/year (CAD) ($761 – $913/year (USD)) [member of a committee with a network-wide mandate that includes more meetings/commitment than a research project]  $1,500/year (CAD) ($1,141/year (USD)) [member of a network steering or executive committee]  $2,000/year ($1,522/year (USD)) [for a 2-3 year project to participate in monthly meetings, to provide project input frequently, and to review work plans, papers, etc. (personal experience of the authors)] | Pay per activity completed or meeting attended |
| Smith, E | $50 (USD)/ hourly  When patient partners occupy more stable and long-term positions within a research team and/or their involvement is related to specific research tasks (e.g., recruitment, data analysis), their salary has to follow established university pay standards | N/R |
| National Institute for Health and Care Research  UCL Partners | £12.50 ($19 (USD)) For involvement in a task or activity such as reading and commenting on an abstract which equates to less than half an hour. For example, reviewing papers for the development of Alerts.  £25 ($38 (USD)) For involvement in a task or activity requiring little or no preparation and which equates to approximately one hour of activity or less. For example, participating in a focus group to provide feedback on a proposal.  £50 ($76 (USD)) For involvement in a task or activity likely to require some preparation and which equates to approximately two hours of activity. For example, a teleconference with related papers to read or review a few short documents.  £60-75 per interview ($91 - $86 per interview (USD)) [Peer researchers/interviewers]  £75 ($86 (USD)) For involvement in a task or activity where preparation is required and which equates to approximately half a day’s activity. For example, participating in a meeting to interview a small number of candidates who have applied to join a committee or panel, participating in a focus group, or delivering training.  £150 ($173 (USD)) For involvement in all-day meetings. For example, attending a committee or panel meeting as an observer prior to becoming an active public member of a committee/panel.  £300 ($346 (USD)) For involvement in all-day meetings that require substantial preparation. For example, when chairing or co-chairing a meeting or when carrying out other discretionary work, which requires additional responsibilities.  £150.00 ($173 (USD)) For attending a full day of training or event organized by NIHR programs.  £50 ($57 (USD)) (document review <50 pages), £125 ($144 (USD)) [51-200 pages], £200 ($230 (USD)) {201-400], £300 ($346 (USD)) [401+] document reviewal  ‘Budgeting for Involvement’ from INVOLVE, which includes an online cost-calculator <https://www.invo.org.uk/resource-centre/payment-and-recognition-for-public-involvement/involvement-cost-calculator/#1> | Pay per activity completed or meeting attended |
| Newfoundland and Labrador's Support for People and Patient-Oriented Research and Trials Unit | $ 200 per annum (CAD) ($152 per annum (USD)) [This is the standard honorarium offered for preparation, attendance at and follow up activities from the Patient Advisory Council meetings and/or Indigenous Council meetings.]  $50 per half day (CAD) (up to 3 hours) ($38 per half day (USD)), $100 per full day (CAD) (3 hours and up) ($57 (USD)) up to max $300 per annum (CAD) ($228 per annum) (USD)) | One payment per year |
| NHS England | £150 per day (more than four hours) ($173 per day (USD)) or £75 per half day (four hours or less) ($86 per half day (USD)). | N/R |
| Rising Tide Foundation | 55-100 (EUR) as compensation for the work of patient experts on review panels or clinical research projects. ($55 - $99 (USD)) | N/R |
| Saskatchewan Centre for Patient-Oriented Research (SCPOR) | $25/hour (CAD) ($19/hour (USD)) | N/R |
| Sepsis Canada | $25/hour (CAD) ($19/hour (USD)) | N/R |
| SPOR Evidence Alliance | $25/ hour (CAD) ($19/hour (USD)) | Pay per activity completed or meeting attended |
| SPOR Networks in Chronic Diseases and the PICHI Network | $25.00 (CAD) ($19 (USD))  $500 to $800 per year (CAD) ($380 to $609 per year (USD)) depending on frequency and numbers of meetings and other factors deemed important by the team (Availability by email; willing and able to participate in a few meetings by phone or in person)  $1000 to $1200 per year (CAD) ($761 – $913/year (USD)) depending on frequency and numbers of meetings and other factors deemed important by the team (Commitment to a committee or group (includes meetings, follow- up actions, etc.))  $1500/year (CAD) ($1,141/year (USD)) Contributing member in a governing committee (includes meetings, follow-up actions, etc.) | Pay per activity completed or meeting attended  Quarterly |
| The Canadian Donation and Transplantation Research Program (CDTRP) | $50 per hour (CAD) ($38/hour (USD)) maximum remuneration is $1500 per year (CAD) ($1,141/year (USD)) | Quarterly |
| The Change Foundation | Minimum wage in Ontario ($15.50/hour (CAD))^a^ ($12/hour (USD)) | N/R |
| The National Health Council | Fair Market Value calculator: https://nationalhealthcouncil.org/fair-market-value-calculator/ | N/R |
| University of Calgary | $25/hour (CAD) ($19/hour (USD)) | N/R |
| US Department of Veteran Affairs | Veteran Consultant Network, $25/hour (USD)  Veteran Engagement Group member, $50/meeting (USD),  Veteran Engagement Group member, $599/yearly (USD) | Quarterly  Monthly |

Currency conversions are based on rates in September 2022

^a^Minimum wage in Ontario in September 2022

**Appendix 7. Population specific compensation details**

| **Population** | **Important compensation considerations** |
| --- | --- |
| Indigenous peoples including Elders, Knowledge Keepers, and other traditional leaders and gatherers | - Performing a traditional cultural service (i.e., opening and closing prayers, Welcome to Territory, Smudging, facilitating a Sharing/Talking/Healing Circle and other ceremonies): $150-200 per service (CAD) ($114 - $152 per service (USD)) - Attendance at a whole day event $400-500 per whole day event (CAD) ($304 - $380 per whole day event (USD)). - When offering honoraria, it should not be viewed as a payment for service, but rather as a gift exchange for knowledge, ceremonies, or blessings. Traditional Indigenous and cultural knowledge is not and cannot be purchased or owned by an individual or institution. - It is particularly important that the honorarium is available at the time of the event and presented prior to the activity in which the Elder has been invited to conduct or participate. - Elders must never be asked to sign a “receipt” as acknowledgement of their gift even if it is financial nor should they be asked for their Social Insurance Number (SIN), or their birth dates. - Additional costs incurred by the Elder, such as parking, mileage, meals and accommodations, must be reimbursed. - Appropriate gifts include tea, blanket, or scarf. |
| Youth and children | - Proxy payment such as vouchers can be offered as recognition for involvement, especially for one-off events or consultations instead of cash payments. - Authorship is highly valued by some youth patient partners. - Cover expenses in advance (such as to a travel company for rail tickets) to avoid young people having to claim reimbursement. If youth partners need to be reimbursed, ensure that any reimbursed expenses are processed quickly and easily. - Consider the costs incurred by an accompanying adult or family member, or those of a support worker, when appropriate - Be mindful of the legal restrictions that limit the times and number of hours that children and young people aged under 16 can undertake as paid activity. - Be aware that young people of working age may be in paid employment, or in receipt of welfare benefits. In these circumstances, the same considerations apply as for adults with regards to tax and benefits. |
| Seldom heard and marginalized populations (i.e., members of LGBTQ+, homeless people, sex workers, individuals living with mental health and/or substance abuse, Traveller communities) | - For people who face multiple barriers caused by marginalization, stigma, and criminalization, equitable compensation ensures that participating in engagement is as barrier-free and inclusive as possible. - When offering co-authorship, consider that patient partners may have more sensitivity about being known publicly as having a health condition or health experience - Particular care should be taken to understand patient partner circumstances for example, pre-booking travel on their behalf so that they are not out-of-pocket. - Consider reimbursing expenses or offering financial compensation in cash as some individuals may not have a bank account |
| Individuals with disabilities | - Monetary value of financial compensation may be increased for patient partners with disabilities - Some people living with disabilities may have a personal assistant to support them to get actively involved in research. Consider the cost of personal assistant attendance. - Recognize that patient partners may be in receipt of state benefits and accepting financial compensation may put their benefit entitlement in jeopardy. |
| Individuals with chronic conditions | - Consider offering financial compensation to patient partners living with chronic disease. They may have to stop paid work and may have increased costs due to additional medical care, childcare or other support needs. If only travel costs and some other expenses are reimbursed for patient involvement, this is usually insufficient and will lead to a very limited availability of patients who may be able to contribute. |

Currency conversions are based on rates in September 2022

**Appendix 8. Items to consider when offering financial compensation to patient partners**

| **Item to consider** | **Number of studies** |
| --- | --- |
| Patient partners can refuse financial compensation payments or choose to accept less than what is offered. | 19 |
| Organize one-on-one meetings with patient partners to discuss financial compensation at the beginning of the project.   - It is the researcher’s responsibility to initiate the conversation around financial compensation. - Be open and transparent about compensation during this meeting and be sure to continue communication throughout the project. - Use the discussion to guide drafting a budget with anticipated hours and financial compensation details. - Patient partners must approve the final budget. | 17 |
| Payment frequency, monetary value and compensation method (i.e., cheque, direct deposit, gift card etc.) should be informed by patient partner preferences.   - Ensure payments are made on time as late payments can undermine respect - If paying cash, offer $20 bills as some businesses don't accept higher - Cheques can take a long time to process and can only be accepted by people with bank accounts. - Offering gift cards without that being the patient partners preference can be patronizing (i.e., dictating where they can spend the money) | 16 |
| Financial compensation is considered taxable income. Thus, payments for involvement are subject to income tax   - In Canada, a T4 is issued for honoraria of $500 (CAD) per year or more. Canada Pension Disability requires disclosure of compensation above a specific amount (recently $5,500). - You may need to collect patient partner social security numbers. Confidentiality measures may be necessary to store this information. - Patient partners who are in receipt of income from medical insurance or as disability payments should be aware that accepting additional compensation can jeopardize future payments. | 15 |
| Budget adequately for compensation before engagement starts   - If engagement is more than budgeted, prepare to pay more. - If engagement is less than budgeted, pay what you promised. - INVOLVE recommends 5% of the project budget should go towards supporting patient engagement. | 10 |
| The value of financial compensation should not reflect the “market value” of patient contributions rather the level of responsibility, type of work, degree of participation, expertise and time committed to the research project   - Although different levels of engagement may require different rates, payments are additive. For example, if a patient partner is engaged at two levels, they should receive both payments. | 8 |
| Compensation rates should be consistent within an institution. For example, patient partners in the same role are compensated the same amount. | 6 |
| Developing a contract or policy a priori can be helpful in outlining engagement activities, expectations, and compensation details. | 3 |
| Review your organizations policy or procedure for financial compensation payments before you develop a contract or budget.   - For example, some organizations cap compensation amounts and anything that exceeds this value will require a contract/salary. | 2 |
